# Facile and Low-Cost Fabrication of SiO_2_-Covered Au Nanoislands for Combined Plasmonic Enhanced Fluorescence Microscopy and SERS

**DOI:** 10.3390/nano13192729

**Published:** 2023-10-08

**Authors:** Alejandro Vidal, Sergio Molina-Prados, Ana Cros, Núria Garro, Manuel Pérez-Martínez, Raquel Álvaro, Gadea Mata, Diego Megías, Pablo A. Postigo

**Affiliations:** 1 Instituto de Micro y Nanotecnología de Madrid (IMN-CSIC), Tres Cantos, 28760 Madrid, Spain; alejandro.vidal@csic.es (A.V.); raquel.alvaro@imm.cnm.csic.es (R.Á.); 2 GROC-UJI, Institut de Noves Tecnologíes de la Imatge (INIT), Universitat Jamue I, 28760 Tres Cantos, Spain; molinapr@uji.es; 3 Institut de Ciència dels Materials (ICMUV), Universitat de València, 46071 Valencia, Spain; ana.cros@uv.es (A.C.); nuria.garro@uv.es (N.G.); 4 Confocal Microscopy Unit, Centro Nacional de Investigaciones Oncológicas (CNIO-ISCIII), 28029 Madrid, Spain; mperez@cnio.es (M.P.-M.); gmata@cnio.es (G.M.); dmegias@cnio.es (D.M.); 5 The Institute of Optics, University of Rochester, Rochester, New York, NY 14627, USA

**Keywords:** Au nanoislands, nanoplasmonic waves, localized surface plasmon resonance, confocal microscopy, fluorescent beads, surface-enhanced Raman scattering, shell-isolated SERS

## Abstract

An easy and low-cost way to fabricate monometallic Au nanoislands for plasmonic enhanced spectroscopy is presented. The method is based on direct thermal evaporation of Au on glass substrates to form nanoislands, with thicknesses between 2 and 15 nm, which are subsequently covered by a thin layer of silicon dioxide. We have used HR-SEM and AFM to characterize the nanoislands, and their optical transmission reveals strong plasmon resonances in the visible. The plasmonic performance of the fabricated substrates has been tested in fluorescence and Raman scattering measurements of two probe materials. Enhancement factors up to 1.8 and 9×10^4^ are reported for confocal fluorescence and Raman microscopies, respectively, which are comparable to others obtained by more elaborated fabrication procedures.

## 1. Introduction

Low-cost nanoparticles are vital in various fields, such as medicine, environmental protection, and energy, due to their unique properties that are not found in bulk materials. They also offer efficient waste treatment and pollution control solutions and contribute to clean energy sources [1]. Low-cost nanoparticles are essential in plasmonic and Surface-Enhanced Raman Scattering (SERS) sensing, offering increased sensitivity and a broad range of applications, including medical diagnostics [2] and environmental monitoring [3,4]. Facile fabrication, the simplified production process, enhances the advantages of these nanoparticles by reducing costs [5,6], allowing rapid production [7], ensuring scalability [8,9], enhancing accessibility [10], and promoting innovation [11]. Low-cost nanoparticles and facile fabrication pave the way for accessible, affordable, and advanced plasmonic and SERS sensing technologies, fulfilling diverse applications across various industries [12]. Metallic nanoplasmonic substrates have already been shown to enhance electromagnetic signals nearby, such as surface-enhanced Raman spectroscopy (SERS) [13,14], metal-enhanced fluorescence (MEF) [15], or plasmonic resonance energy transfer-based nano-spectroscopy [16]. SERS generally relies on hot spots localized in gaps between plasmonic nanoparticle assemblies, also known as SERS substrates [17]. The operating basis of these nanoplasmonic substrates is a phenomenon known as localized surface plasmon resonance (LSPR), which originated as a result of the interaction between electromagnetic fields (light) and surface-free charges (electrons) from metallic structures. LSPR is attributed to the collective oscillation of free electrons in metallic nanomaterial surfaces due to light incidence of a specific wavelength, giving rise to unique properties. LSPR is interesting for optics applications and other fields like solar cells [18] or biological sensors with higher sensitivity due to signal enhancement [19,20]. LSPR properties of a surface depend on the interface materials, size, geometrical distribution, and even on the spacing distance between nanostructures. Those parameters themselves are highly dependent on the nanofabrication method followed. The latest studies have shown that metallic nanoislands can show LSPR effects tuned for different wavelength emissions, leading to an amplification of signals such as SERS and MEF that are maximized when LSPR wavelength suits the wavelength of the incident light [21]. Monometallic nanostructured substrates show LSPR effects under the presence of light. Bimetallic alloys have raised interest in this field due to the possibility of tuning the surface plasmon resonance wavelength [22,23,24,25]. Au/Ag alloy-made nanoislands have been shown to vary in LSPR wavelength, depending on the concentration of Au and Ag present in the mixture, from 450 to 680 nm, going red-shifted as the proportion of Au increases [23,24,25]. The latest approaches have focused on the fabrication of nanoislands made of bimetallic alloys of Au and Ag by depositing thin layers of these materials in the proportion wanted and fusing these nanolayers to form isolated nanoislands through a solid dewetting process of thermal annealing [22,23,24,25]. Lately, it has also been shown that the presence of discrete Au nanoislands in the wall of plasmonic nanocapsules makes them suitable for photothermal therapies of melanoma tumors, thus providing the possibility to develop imaging-guided photothermal probes [26].

In this work, we have fabricated Au nanoislands with plasmonic properties using an easy and low-cost method. The Au is evaporated on glass substrates by electron beam evaporation, and the nanoislands are directly formed. Afterwards, a thin silicon dioxide (SiOx) layer is deposited by plasma-enhanced chemical vapor deposition (PECVD). We have measured the strong plasmonic absorption for each type of nanoisland, which differ in the thickness of evaporated Au, ranging from 2 to 15 nm. We found that the Au thickness can tune the plasmon resonance in a wide range (100 nm). Finally, we used the samples as substrates for plasmonic-enhanced fluorescent emission of microspheres and SERS of a dye molecule. Fluorescence-confocal microscopy provides enhancements up to 1.8 times, similar to the obtained by other more elaborated methods [25]. Regarding SERS sensing, these substrates, with the plasmonic cores isolated by the thin silica layer, do not modify the analyte by preventing direct adsorption onto the metal [27]. This can be of paramount importance when sensing species that may undergo electrochemical reactions [28]. Enhancement factors up to 9 × 10^4^ have been computed for a molecular dye probe.

## 2. Materials and Methods

### 2.1. Substrate Fabrication

**Figure 1:**
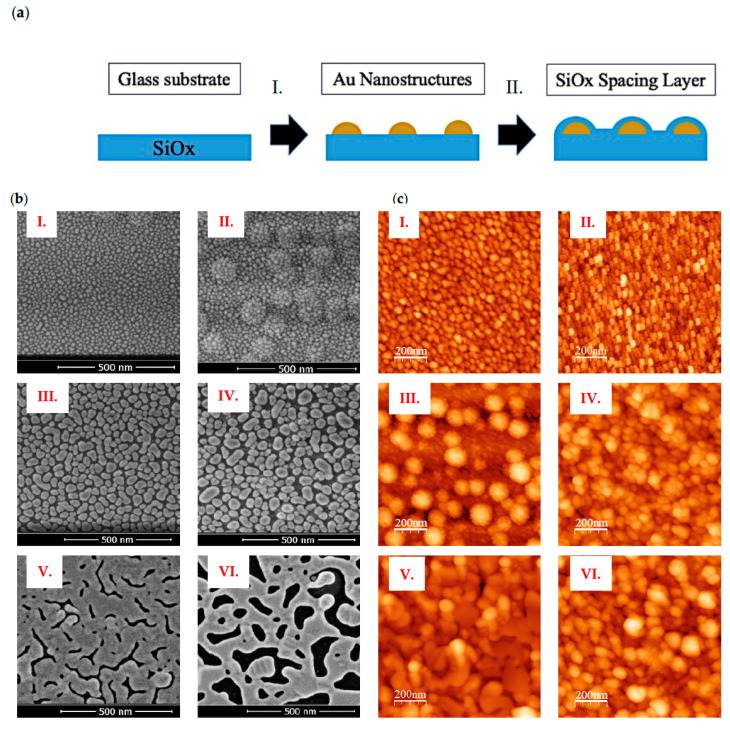
(**a**) Schematic illustration of Au nanoislands fabrication: I. Electron beam Au evaporation. II. PECVD SiOx deposition; (**b**) SEM topography images (secondary electrons); (**c**) AFM (tapping mode) images. I. 2 nm Au, 2 nm SiOx II. 2 nm Au, 5 nm SiOx III. 7 nm Au, 2 nm SiOx IV. 7 nm Au, 5 nm SiOx V. 15 nm Au, 2 nm SiOx VI. 15 nm Au, 5 nm SiOx. The maximum AFM heights (white color) for each sample are: I. 14.44 nm; II. 6.25 nm; III. 41.10 nm; IV. 44.67 nm; V. 71.96 nm; VI. 43.77 nm. The RMS roughness values are: I. 1.6 nm; II. 0.85 nm; III. 7.32 nm; IV. 6.78 nm; V. 7.25 nm; VI. 46.49 nm. The average heights are: I. 5.43 nm; II. 2.74 nm; III. 14.53 nm; IV. 20.27 nm; V. 24.30 nm; VI. 17.90 nm.

Au monometallic nanoislands substrates were fabricated using a simple method composed of two deposition steps on circular glass substrates 0.1 mm thick and 13 mm in diameter (Figure 1a). The first step involved depositing a thin layer of Au on top of the glass substrate using an electron beam evaporator. Thicknesses of 2, 7, and 15 nm of Au were chosen, with deposition rates varying from 2 Å/s for 2 and 7 nm thick to 0.5 Å/s for 15 nm thick. Our laboratory previously observed that using an electron beam evaporator with small metal thicknesses (less than 25 nm) led to an aggregation of Au particles around specific nucleation points rather than a homogeneous distribution along the whole surface. The observed color of these Au nanofilms varied with the thickness deposited, ranging from light pink at 2 nm width, dark blue at 7 nm, and dark yellow at 15 nm, suggesting different optical properties. Subsequently, a thin film of SiOx was deposited on top of the Au layer using plasma-enhanced chemical vapor deposition with Surface Technology Systems 310PC-DF capacitive plasma equipment operating at 20 W power, 300 °C temperature, and SiH_4_ and N_2_O as precursor gases. This SiOx deposition aimed to create a spacing layer between the Au nanoparticles and the surrounding media to avoid surface plasmon quenching. We chose deposited thicknesses of 2 and 5 nm of SiOx combined with the three Au thicknesses previously mentioned to observe possible differences during fluorescent microscopy measurements. Sample morphology characterization was performed initially using scanning electron microscopy (SEM). Images were taken using a SEM FEI VERIOS 460 with EDX (energy-dispersive X-ray spectroscopy) at 2 kV and 13 pA current. Figure 1b shows how the Au deposition acquires a nanoislands-like appearance without further thermal dewetting processes needed. Fully isolated Au nanoislands can be identified when depositing 2 nm of Au, whereas the nanoislands fuse to form broader structures when increasing up to 15 nm. EDX analysis confirmed the presence of Au in the samples. The area fraction occupied by Au nanoparticles was computed from SEM images using ImageJ (Fiji) [29]. Au thickness of 2 nm led to 44% area fraction occupied, similar to 7 nm with an area of around 48%, whereas 15 nm of Au was close to 90% area covered. Additionally, atomic force microscopy (Bruker—Dimension Icon AFM) images were taken to evaluate the 3D structure and appearance of these nanostructures (Figure 1c) in tapping mode (1 × 1 µm area). See Figure S1 in Supplementary for AFM profiles.

### 2.2. Substrates Characterization

**Figure 2:**
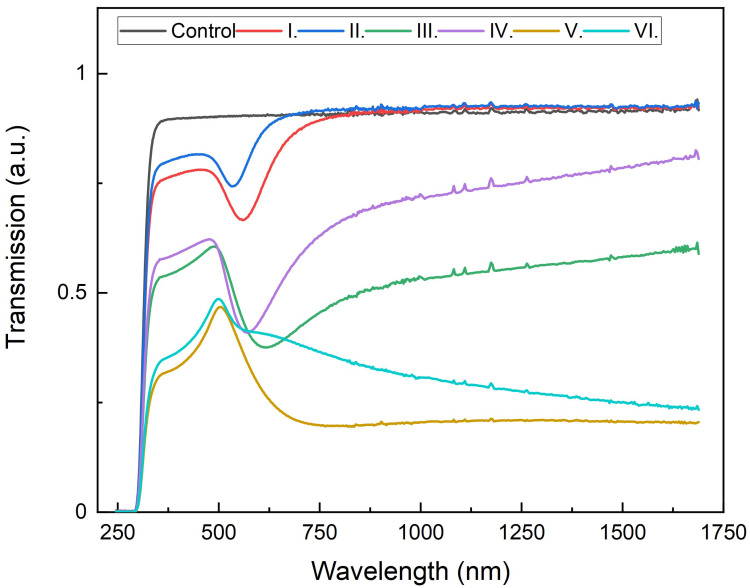
Light transmission of the fabricated nanoislands depending on wavelength. Legend: I. 2 nm Au, 2 nm SiOx; II. 2 nm Au, 5 nm SiOx; III. 7 nm Au, 2 nm SiOx; IV. 7 nm Au, 5 nm SiOx; V. 15 nm Au, 2 nm SiOx; VI. 15 nm Au, 5 nm SiOx; Control: glass coverslip.

Transmission experiments were carried out in an ellipsometer at normal incidence. Figure 2 shows light transmission versus the wavelength for each of the substrates (I. 2 nm Au, 2 nm SiOx; II. 2 nm Au, 5 nm SiOx; III. 7 nm Au, 2 nm SiOx; IV. 7 nm Au, 5 nm SiOx; V. 15 nm Au, 2 nm SiOx; and VI. 15 nm Au, 5 nm SiOx). The shape of the curve is very similar for all substrates, going from no transmission in the ultraviolet to a sharp increase until reaching the first peak at around 500 nm, and then follows a plasmonic-related decrease and a boost for red wavelengths until a plateau is reached for the infrared. As the proportion of Au increases, the overall transmission decreases, showing sharper intensity decay in the infrared.

Optical characterization was continued by evaluating the change in fluorescence intensity using fluorescence confocal microscopy. For that purpose, fluorescent PS-Speck green microspheres were used, with a diameter of 0.175 ± 0.005 μm and excitation/emission wavelength maxima of 505/515 nm [30]. A drop of these fluorescent spheres was added to each fabricated substrate, plus simple glass substrates that served as controls. At least five z-stacks of different microspheres in different fields of view were acquired for each substrate in a Leica DMI6000B wide-field microscope equipped with a 20× 0.5NA objective and A4, L5, and N2.1 fluorescence filter cubes (Leica-Microsystems). Final images were obtained for quantification by performing the maximum projection of the z-stacks with v2.6 LAS AF software. Each picture contained hundreds of beads; consequently, a macro for ImageJ (Fiji) [29] was implemented to localize the beads and compute the mean intensity considering their whole volume.

The SERS performance of the substrates was tested using Methylene Blue (MB) as a probe molecule. The set-up consisted of a Jobin-Yvon Xplora Raman confocal microscope equipped with three laser diodes (532, 638, and 785 nm). A drop of 0.1 mM MB solution was added on the fabricated substrates and performed 11-point SERS scans. All Raman data were acquired in backscattering configuration with a 100× objective, using the 1200 g/mm diffraction grating in a spectral range of 200 to 2000 cm^−1^. The excitation power was 21 kW/cm^2^, and the accumulation time for the spectra was 2 s. Each spectrum was analyzed to calculate the enhancement factor (EF) and its relative variation using computational tools.

## 3. Results and Discussion

**Figure 3:**
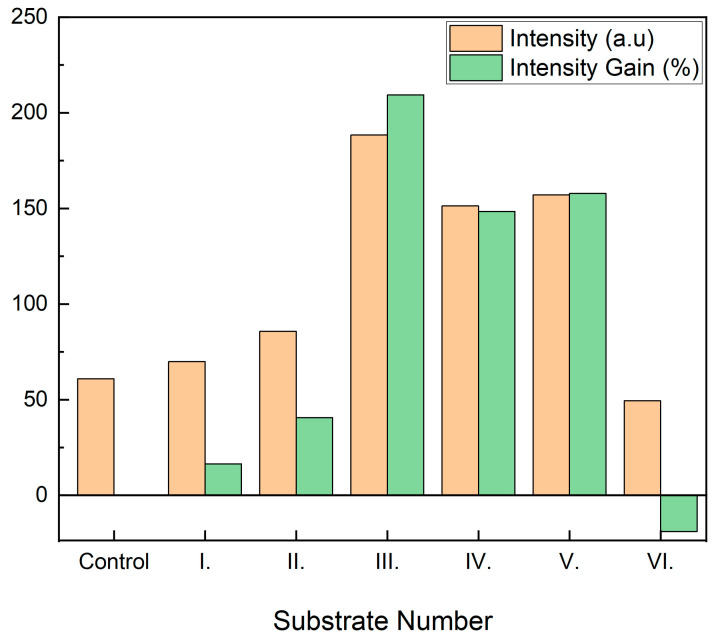
Mean intensity of fluorescent beads under each type of Au substrates in arbitrary units (orange) and gain percentage in mean intensity as compared to the control (green). (I. 2 nm Au, 2 nm SiOx; II. 2 nm Au, 5 nm SiOx; III. 7 nm Au, 2 nm SiOx; IV. 7 nm Au, 5 nm SiOx; V. 15 nm Au, 2 nm SiOx; VI. 15 nm Au, 5 nm SiOx.)

For each sample, the intensity of the beads was computed as previously mentioned, with results displayed in Figure 3. The mean intensity (a.u.) corresponds to the orange bar, whereas the green bar corresponds to the percentage increase in mean intensity from simple glass control to Au-based substrates. The samples were fabricated and measured several times, showing reproducible results.

**Figure 4:**
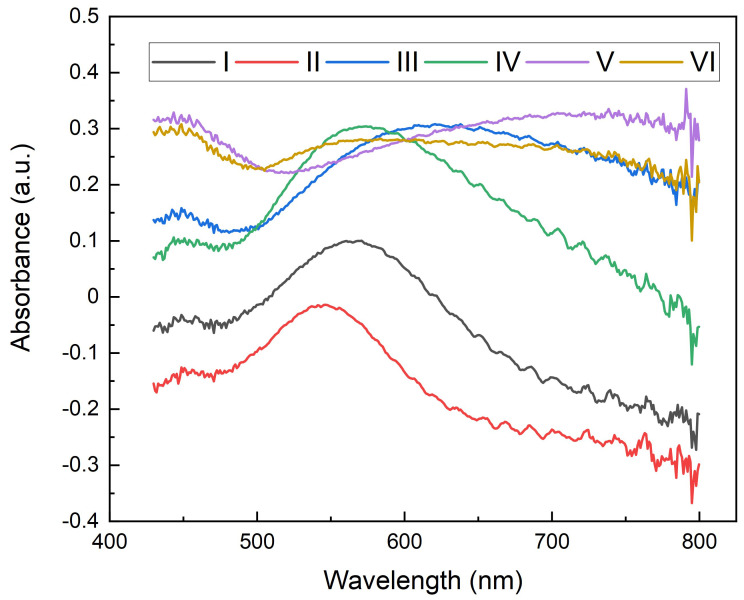
Absorbance of fabricated nanoislands. (I. 2 nm Au, 2 nm SiOx; II. 2 nm Au, 5 nm SiOx; III. 7 nm Au, 2 nm SiOx; IV. 7 nm Au, 5 nm SiOx; V. 15 nm Au, 2 nm SiOx; VI. 15 nm Au, 5 nm SiOx.)

Intensity gain is almost generalized, except for substrate VI, where a slight decrease of around 19% is observed. Substrates IV and V show a solid increase of about 150%. In comparison, the sharpest increase corresponds to substrate III with 210%. The 2 nm Au substrates show very weak enhancements, consistent with transmission experiments (Figure 2) since plasmonic transmission peaks are the highest at around 500 nm (working fluorescence wavelength). Regarding SiOx spacing layer in 2 nm Au substrates, 5 nm deposition seems to work better than 2 nm, maybe because of better plasmon confinement, due to having a higher amount of dielectric material surrounding nanoislands. Both 7 nm Au depositions show solid enhancements with low transmission peaks at 500 nm; 2 nm SiOx spacing layer seems more effective because the transmission peak is closer to the working fluorescence wavelength (Figure 2). Regarding 15 nm Au substrates, the morphologic distribution of the gold layer is much different since the nanoislands are fused (Figure 1b) and diffuse the transmission peak, giving rise to some disparity in the intensity enhancement that cannot be easily explained. As LSPR is due to specific light absorption by the gold nanoislands, we further performed absorption measurements to confirm the plasmonic effects. Absorbance can be obtained using the expression A = 1 − T − R. We measured that the transmission (T) and reflection (R) were at normal incidence using a SENTECH FTP Advanced Reflectometer connected to an OLYMPUS BH-2 optical microscope. As shown in Figure 4, the absorbance of 2 nm-thick gold substrates (I and II) is low for 500 nm wavelengths, which was consistent with low-intensity amplification (Figure 3). Good-performing 7 nm-thick gold substrates (III and IV) led to the highest intensity gains (Figure 3), showing higher absorptions at 500 nm, which may explain the higher intensity amplification for that wavelength. Regarding 15 nm-thick gold substrates, they both show higher absorptions; however, there is also an intensity drop for substrate VI (Figure 3). As previously mentioned, this may be explained by the morphology and characteristics of the gold layer. In any case, absorbance peaks for these gold substrates are around 540 nm wavelength, similar to transmission peaks registered (Figure 2), as one can expect. Negative absorbance values are related to light scattering off the acceptance angle under the microscope.

**Figure 5:**
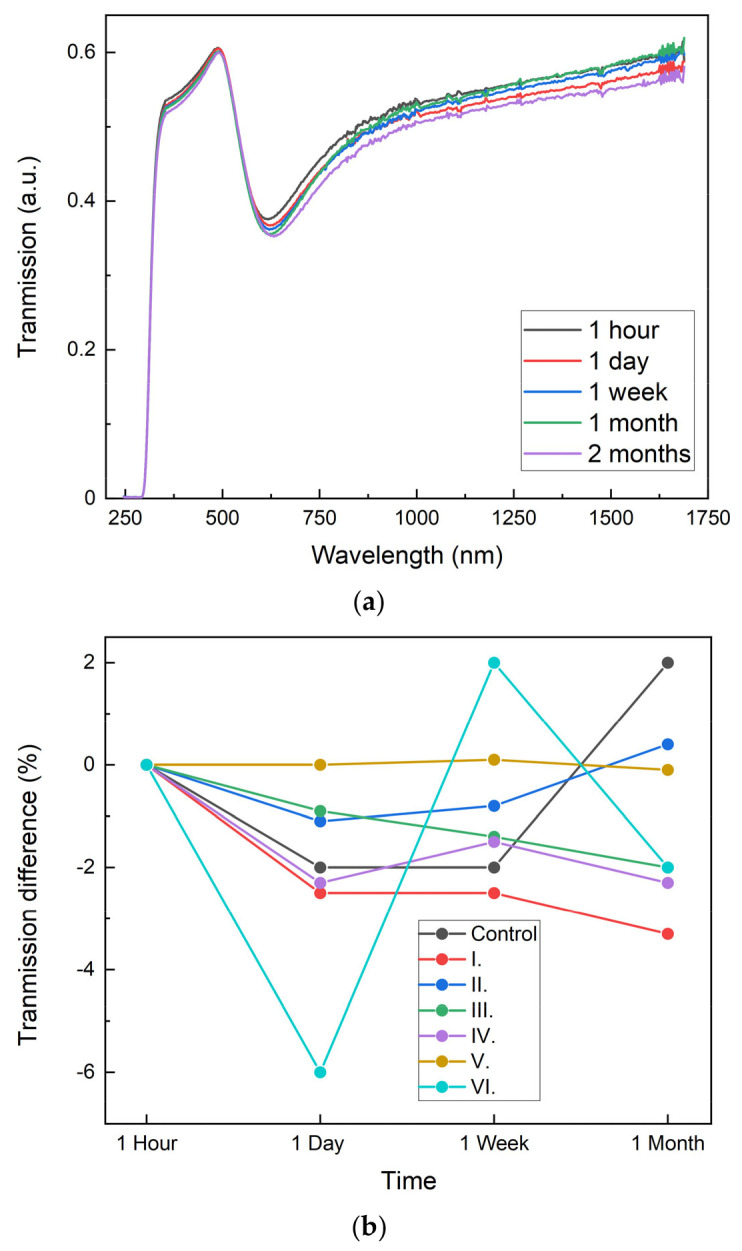
(**a**) Transmission intensity of best working substrate (III) at different times; (**b**) Light transmission difference of substrates at different times as compared to t = 0. (I. 2 nm Au, 2 nm SiOx; II. 2 nm Au, 5 nm SiOx; III. 7 nm Au, 2 nm SiOx; IV. 7 nm Au, 5 nm SiOx; V. 15 nm Au, 2 nm SiOx; VI. 15 nm Au, 5 nm SiOx.)

Additionally, we checked Au stability measuring the transmission intensity over time. Figure 5a shows that the transmission curves almost completely overlap for the best working substrate (III). This means that Au transmission properties are kept for at least one month without significant perturbations. Figure 5b shows a broader view of the transmission change (as a percentage) for all the fabricated samples. If we assume that the error range of the control sample (glass without any nanoislands) of approximately ±2% is similar to the intrinsic error of the ellipsometer, then no substrate shows significant losses, which are even lower for the three best substrates (II, IV, and V). Only substrate VI showed a somewhat unexpected change in the transmission. Consequently, the stability of substrates covered with Au nanoislands seems to be excellent and well-kept over time, a characteristic that we attribute to the effect of the SiOx-protecting layer.

**Figure 6:**
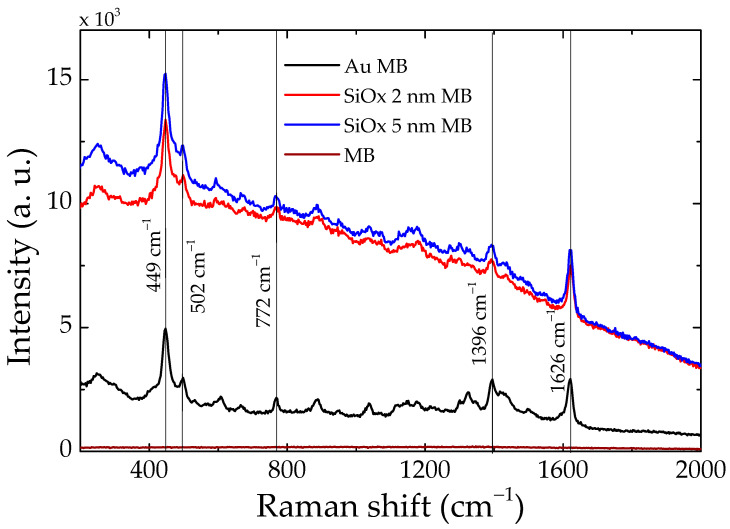
MB Raman spectra study performed on the substrate with 15 nm Au nanoislands. A glass base with MB deposit has been used as a control group. The substrates indicated as SiOx 2 and 5 nm correspond to those used previously, V. and VI. (V. 15 nm Au, 2 nm SiOx; VI. 15 nm Au, 5 nm SiOx.) The spectra shown in the graph are the average spectra obtained in the study.

All samples were tested as SERS substrates for a MB probe using different laser lines. As a matter of fact, wavelength selection is very relevant, as MB has a wide absorption spectrum centered at 650 nm. In order to avoid other resonant processes, we used a wavelength of 785 nm, outside the molecule absorption band. Figure 6 shows the Raman spectra of MB obtained with the molecules deposited either on glass, which is undetectable, or on 15 nm Au substrates, where a very strong signal can be recorded. The central frequency of some of the most intense peaks has been indicated with a vertical line. All these modes can be assigned to well-known vibrations of the MB molecule: 449 and 502 cm^−1^ are skeletal deformations of C-N-C; 772 cm^−1^ is in-plane bending of C-H; 1396 cm^−1^ corresponds to the symmetrical stretching of C-N; and 1626 cm^−1^ is the ring stretching of C-C. The SiOx layer covering the Au nanoislands is not detrimental for SERS; on the contrary, it increases both the Raman signal and the background of the spectra, with the most intense signal being that of the 5 nm SiOx shield. No frequency shifts are introduced by the SiOx layer either. Figures S2 and S3 in Supplementary show additional MB Raman spectra.

The SERS enhancement factor (EF) has been computed for all the studied substrates. This is defined as the ratio between the Raman intensity measured on the SERS substrate $I_{SERS}$ and that of the bare MB molecule measured in solution $I_{NRS}$. These intensities should be normalized by the molecular concentrations both on the surface, $N_{surf}$, and in the solution volume, $N_{vol}$:

$$EF=\frac{{I_{SERS}/N}_{surf}}{I_{NRS}/N_{vol}}$$

We integrated the 449 and 504 cm^−1^ peaks of the MB spectrum, subtracting the background signal, in order not to overestimate the values. For non-resonant Raman scattering, the probed solution volume was estimated as a cylinder with 10 μm radius and 100 μm height. The tested area on the SERS substrate was a circle with a 1 μm radius. Considering the molar concentration of the MB solution 0.1 mM, the tested molecules were $1.9\times 10^{9}$ in solution and $3.8\times 10^{7}$ adsorbed on the SERS substrate. These calculations may underestimate the EF, as we assume that the surface of the SERS substrate is saturated by the MB molecules.

**Figure 7:**
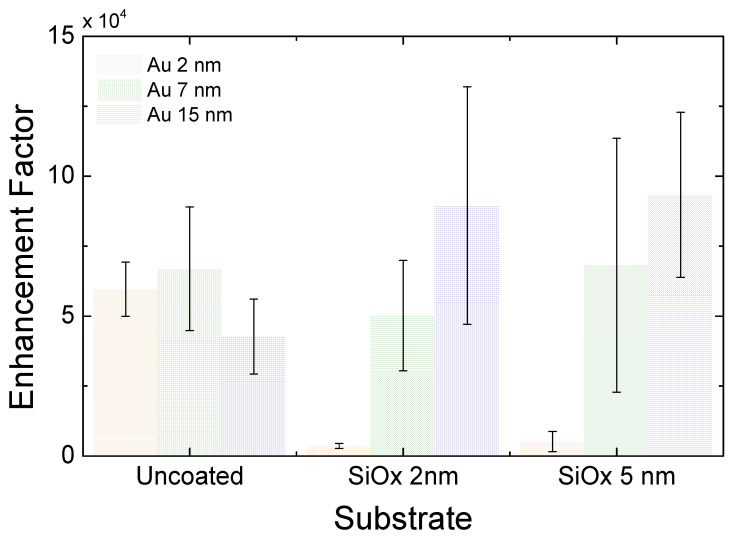
Enhancement factors mean values and standard deviations for different substrates and SiOx shields. The data have been grouped into three categories: uncoated Au nanoislands (2 nm Au, 7 nm Au, and 15 nm Au), substrates coated by 2 nm SiOx (substrates I., III., and V.), and substrates with 5 nm SiOx coatings (substrates II., IV., and IV.) (I. 2 nm Au, 2 nm SiOx; II. 2 nm Au, 5 nm SiOx; III. 7 nm Au, 2 nm SiOx; IV. 7 nm Au, 5 nm SiOx; V. 15 nm Au, 2 nm SiOx; VI. 15 nm Au, 5 nm SiOx).

Figure 7 depicts the EF and their standard deviations obtained from the statistical study of the 99 spectra analyzed, 11 for each kind of substrate. The uncoated Au 2 nm substrates are adequate SERS substrates. However, when a SiOx coating is applied, and the test molecule is moved 2–5 nm away from the substrate surface, the Raman signal decays, and the background signal increases drastically. On the other hand, in the case of Au 7 nm and 15 nm, where SERS performance of the uncoated substrates was as good as that of Au 2 nm, the additional SiOx layers do not quench the EF and can even improve the average statistical values. We observe, however, that SiOx shields tend to increase the EF dispersion from point to point. Taking into account that the effect of the SiOx layer is to move the position of the test molecule away from the plasmonic hot-spots of the substrate, we can explain why for the smaller islands, where hot-spots are more localized, the shield layer reduces the EF, and, for larger islands, the SERS signal is more stable. This is the reason why, for substrates with small nanoislands, the Raman signal decreases drastically, whereas the Raman signal is intensified for 15 nm. Overall, our substrates present reasonable performance and provide comparable or even better figures than SERS commercial substrates [31], both for EF and reproducibility [32,33,34]. SERS stability was also tested. After two months, the SERS measurements were repeated to ensure that the uncoated substrates did not show reduced Raman activity due to ageing. In all cases, the EF values were very similar to those obtained previously. No change in the relative variation in the spectra was observed either.

## 4. Conclusions

In conclusion, we have successfully developed an easy, low-cost method for the nanofabrication of large-area plasmonic substrates covered with Au-nanoislands. Depending on the thickness of the Au deposited, the substrates show plasmonic-tunable properties and a significant increase in the enhancement of green fluorescent intensity with values comparable to the measured in other plasmonic nanostructures fabricated with more complex methodologies. The nanoisland layers show good stability over time that we attribute to the SiOx layer deposited for protection. The results can be helpful for enhanced confocal fluorescent microscopy, where the sometimes-weak fluorescence signal needs to be enhanced by any possible means. Furthermore, all substrates provided surface-enhanced Raman scattering. Those with larger nanoislands and SiOx coatings showed the best performances. The fabricated substrates present enhanced fluorescence during confocal fluorescence microscopy measurements up to 1.8 times, comparable to others obtained by more elaborated fabrication procedures, and an enhancement factor for Raman scattering above 10^4^. For the SiOx-covered Au nanoislands, samples with nominal thicknesses of Au smaller than SiOx have lower enhancement factors. The best enhancement factors are found for nominal Au thickness above 7 nm and smaller SiOx spacings of 2 nm and 5 nm. Substrates remained optimal over time, as there was no degradation–oxidation. Therefore, this nanofabrication method allows for the synthesis of large-area plasmonic substrates with high sensitivity and reproducibility for SERS measurements.

## Data Availability

The data presented in this study is available on request from the corresponding authors.

## Supplementary Materials

The following supporting information can be downloaded at: https://www.mdpi.com/article/10.3390/nano13192729/s1. Figure S1. AFM (tapping mode) images and profiles along the white lines. I. 2 nm Au, 2 nm SiOx II. 2 nm Au, 5 nm SiOx III. 7 nm Au, 2 nm SiOx IV. 7 nm Au, 5 nm SiOx V. 15 nm Au, 2 nm SiOx VI. 15 nm Au, 5 nm SiOx. The maximum AFM heights (white color) for each sample are: I. 14.44 nm; II. 6.25 nm; III. 41.10 nm; IV. 44.67 nm; V. 71.96 nm; VI. 43.77 nm. The RMS roughness values are: I. 1.6 nm; II. 0.85 nm; III. 7.32 nm; IV. 6.78 nm; V. 7.25 nm; VI. 46.49 nm. The average heights are: I. 5.43 nm; II. 2.74 nm; III. 14.53 nm; IV. 20.27 nm; V. 24.30 nm; VI. 17.90 nm. Figure S2. MB Raman spectra study performed on the substrate with 2 nm Au nanoislands. A glass base with MB deposit has been used as a control group. The substrates indicated as SiOx 2, and 5 nm correspond to those used previously I. and II. [I. 2 nm Au, 2 nm SiOx II. 2 nm Au, 5 nm SiOx]. The spectra shown in the graph are the average spectra obtained in the study. Figure S3. MB Raman spectra study performed on the substrate with 7 nm Au nanoislands. A glass base with MB deposit has been used as a control group. The substrates indicated as SiOx 2, and 5 nm correspond to those used previously II. and IV. [II. 7 nm Au, 2 nm SiOx IV. 7 nm Au, 5 nm SiOx]. The spectra shown in the graph are the average spectra obtained in the study.
