# Supplementary material for: Facile and Low-Cost Fabrication of SiO2-Covered Au Nanoislands for Combined Plasmonic Enhanced Fluorescence Microscopy and SERS
Source: Nanomaterials (Basel). 2023 Oct 8;13(19):2729. doi: 10.3390/nano13192729 (PMC10574186; doi:10.3390/nano13192729)
Supplement: Supplementary file 1 [file nanomaterials-13-02729-s001.zip › nanomaterials-2621079-supplementary.pdf]

Facile and Low-cost Fabrication of SiO_2-covered Au Nanoislands for combined Plasmonic Enhanced Fluorescence Microscopy and SERS. Supplementary material

1. AFM Profiles of Samples

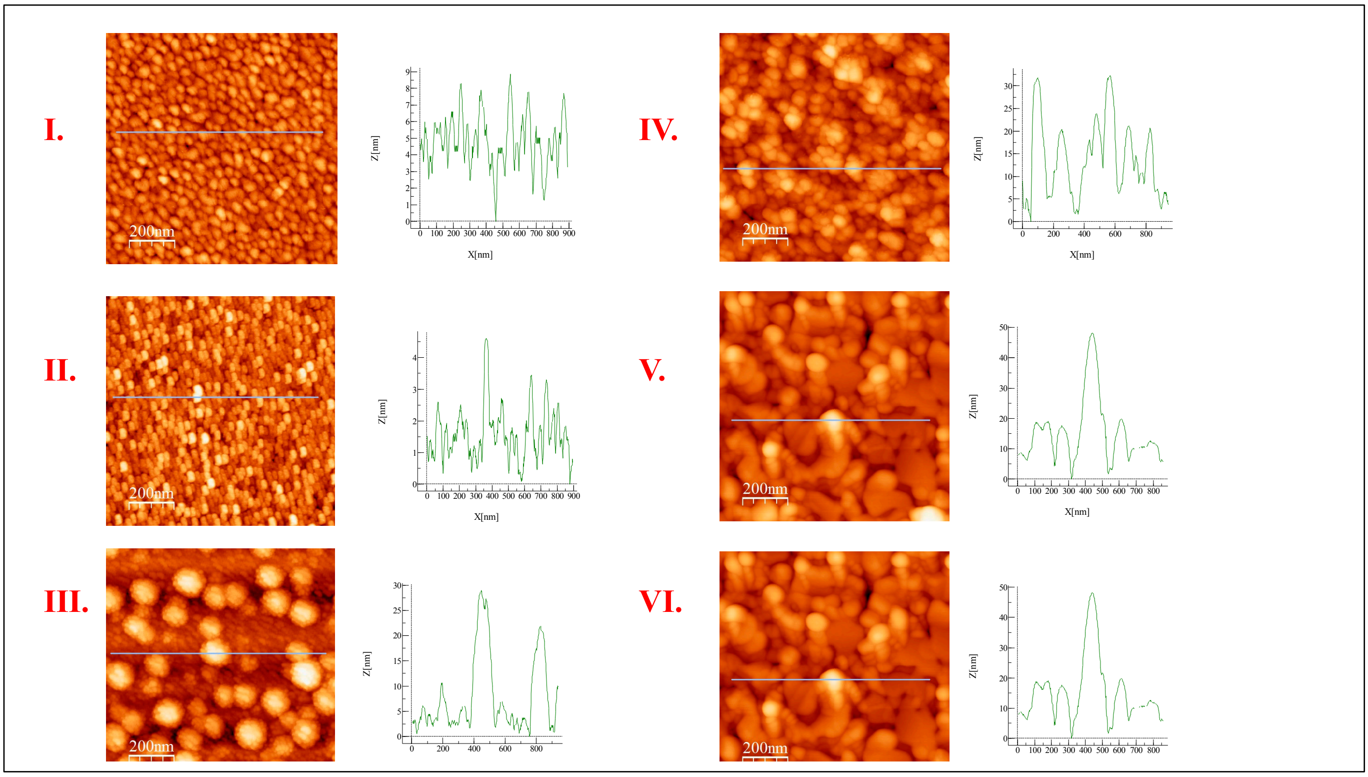

Figure S1. AFM (tapping mode) images and profiles alonf the whote lines. I. 2 nm Au, 2 nm SiOx II. 2 nm Au, 5 nm SiOx III. 7 nm Au, 2 nm SiOx IV. 7 nm Au, 5 nm SiOx V. 15 nm Au, 2 nm SiOx VI. 15 nm Au, 5 nm SiOx. The maximum AFM heights (white color) for each sample are: I. 14.44 nm; II. 6.25 nm; III. 41.10 nm; IV. 44.67 nm; V.71.96 nm; VI. 43.77 nm. The RMS roughness values are: I. 1.6 nm; II. 0.85 nm; III. 7.32 nm; IV. 6.78 nm; V. 7.25 nm; VI. 46.49 nm. The average heights are: I. 5.43 nm; II. 2.74 nm; III. 14.53 nm; IV. 20.27 nm; V. 24.30 nm; VI. 17.90 nm.

2. Raman Spectra

When substrates with a smaller nanoisland size were used, the data obtained are notably different. In the first case, 2 nm nanoisland in size, dropping the MB on the substrate produces that the Raman signal obtained is shielded by the SiOx layer at both 2 nm and 5 nm thickness. The reduction of the signal is such a big that it is only possible to identify the 3 peaks of highest intensity of the MB, shown in Figure S1. For the 7 nm Au substrates, the MB spectrum is clearly identifiable for both the 2 nm and 5 nm SiOx coated and uncoated substrates. As depicted in Figure S2, increasing the size of the SiOx coating increases the background signal of the spectrum, but does not increase the identifiable spectrum of the MB. Another problem generated by the coating on the 7 nm Au nanoislands is the increase of the variation between spectra, i.e., the substrate without coating shows practically no variation since all the signals are practically the same, while for the substrates with SiOx the variation generates that the Raman signal changes significantly in different positions.

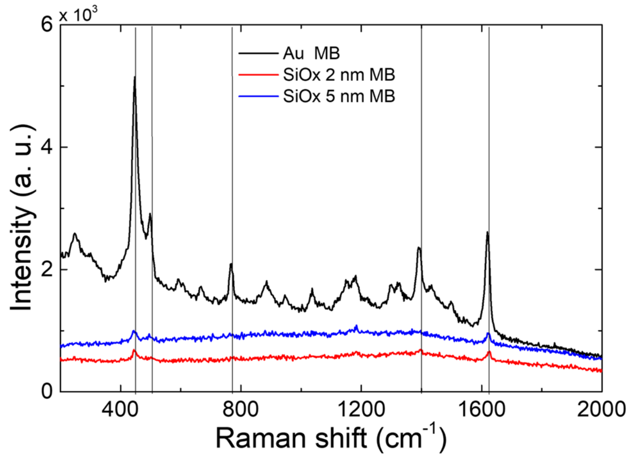

Figure S2. MB Raman spectra study performed on the substrate with 2 nm Au nanoislands. A glass base with MB deposit has been used as a control group. The substrates indicated as SiOx 2, and 5 nm correspond to those used previously I. and II. [I. 2 nm Au, 2 nm SiOx II. 2 nm Au, 5 nm SiOx]. The spectra shown in the graph are the average spectra obtained in the study.

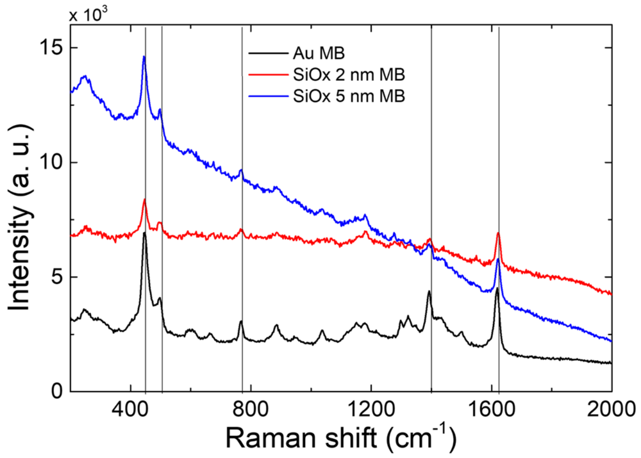

Figure S3. MB Raman spectra study performed on the substrate with 7 nm Au nanoislands. A glass base with MB deposit has been used as a control group. The substrates indicated as SiOx 2, and 5 nm correspond to those used previously II. and IV. [II. 7 nm Au, 2 nm SiOx IV. 7 nm Au, 5 nm SiOx]. The spectra shown in the graph are the average spectra obtained in the study.
